# Changes in quantity plant-based protein intake on type 2 diabetes remission in coronary heart disease patients: from the CORDIOPREV study

**DOI:** 10.1007/s00394-022-03080-x

**Published:** 2023-03-04

**Authors:** Francisco M. Gutierrez-Mariscal, Juan F. Alcalá-Diaz, Gracia M. Quintana-Navarro, Silvia de la Cruz-Ares, José D. Torres-Peña, Magdalena P. Cardelo, Antonio P. Arenas-Larriva, María M. Malagón, Juan L. Romero-Cabrera, José M. Ordovás, Pablo Pérez-Martínez, Javier Delgado-Lista, Elena M. Yubero-Serrano, José Lopez-Miranda

**Affiliations:** 1 Lipids and Atherosclerosis Unit, Unidad de Gestión Clinica Medicina Interna, Maimonides Institute for Biomedical Research in Córdoba (IMIBIC), Reina Sofia University Hospital, University of Córdoba, Av. Menendez Pidal, S/N. 14004, Córdoba, Spain; 2 CIBER Physiopathology of Obesity and Nutrition (CIBEROBN), Institute of Health Carlos III, Madrid, Spain; 3 Food Science and Technology Department, Universidad de Córdoba, Darwin Building, 14014 Córdoba, Spain; 4 Department of Cell Biology, Physiology and Immunology, University of Cordoba, Córdoba, Spain; 5 Jean Mayer US Department of Agriculture Human Nutrition Research Center on Aging, Tufts University School of Medicine, Boston, MA USA; 6 IMDEA-Food Institute, CEI UAM + CSIC, Madrid, Spain

**Keywords:** Plant protein, Diabetes Remission, Mediterranean diet, Dietary pattern

## Abstract

**Purpose:**

Diabetes remission is a phenomenon described in the context of drastic weight loss due to bariatric surgery or low-calorie diets. Evidence suggests that increasing the intake of plant protein could reduce the risk of type 2 diabetes. We sought for association between changes in plant protein intake in the context of 2 healthy diets without weight loss nor glucose-lowering medication, and diabetes remission in coronary heart disease patients from the CORDIOPREV study.

**Methods:**

Newly diagnosed type 2 diabetes participants without glucose-lowering treatment were randomized to consume a Mediterranean or a low-fat diet. Type 2 diabetes remission was assessed with a median follow-up of 60 months according to the ADA recommendation. Information on patient's dietary intake was collected using food-frequency questionnaires. At first year of intervention, 177 patients were classified according to changes in plant protein consumption into those who increased or decreased its intake, in order to perform an observational analysis on the association between protein intake and diabetes remission.

**Results:**

Cox regression showed that patients increasing plant protein intake were more likely to remit from diabetes than those who decreased its intake (HR = 1.71(1.05–2.77)). The remission occurred mainly at first and second year of follow-up with diminished number of patients achieving remission in the third year onwards. The increase in plant protein was associated with lower intake of animal protein, cholesterol, saturated fatty acids, and fat, and with higher intake of whole grains, fibre, carbohydrates, legumes, and tree nuts.

**Conclusion:**

These results support the need to increase protein intake of vegetal origin as dietary therapy to reverse type 2 diabetes in the context of healthy diets without weight loss.

**Supplementary Information:**

The online version contains supplementary material available at 10.1007/s00394-022-03080-x.

## Introduction

The prevalence of type 2 diabetes mellitus (T2DM) is steadily increasing worldwide with a consequence socioeconomic burden on health services, the economy and society [1, 2]. Moreover, when it is presented as comorbidity along with coronary heart disease (CHD) the risk for vascular complications increases. Therefore, the prevention of T2DM should be a high priority in cardiovascular disease patients.

It has recently been demonstrated that T2DM is reversible in patients who undergo bariatric surgery for obesity [3–5] or follow moderate energy restriction diets [6], counteracting the fat accumulation in liver and pancreas. In this context, DiRECT (Diabetes REmission Clinical Trial) study has demonstrated that a dietary intervention with a liquid formula calorie restriction diet achieved T2DM remission, in a population of patients with recent diagnosed disease, after a drastic weight loss in 2 years follow-up [7, 8]. In the CORDIOPREV (CORonary Diet Intervention with Olive oil and cardiovascular PREVention) study population, we have previously described a set of newly diagnosed diabetic patients who achieved T2DM remission, in a follow-up of 5 years, after the consumption of two healthy diets (a Mediterranean diet or a low-fat) without weight loss nor pharmacological treatment [9, 10]. However, besides the influence of previous status in insulin resistance and beta-cell functionality on T2DM remission rates, we did not find differences among both diets.

Plant protein-based diets have been proposed in order to improve diabetes-related parameters such as glucose metabolism control and glycated haemoglobin (HbA1c). Therefore, this dietary strategy could be appropriated in the management of diabetic patients and in the prevention of the disease [11]. In this regard, we have recently reported that changes in protein consumption toward an increment in plant protein-based foods presented less probability of T2DM onset in non-diabetic patients from CORDIOPREV study [12].

Based on these premises, the objective of this work was to evaluate, within the framework of the CORDIOPREV study, whether changes in dietary habits towards the consumption of increasing plant-based proteins, at expense of diminishing proteins from animal sources, were associated with the increase of T2DM remission in a population of newly diagnosed diabetic patients with CHD without weight loss nor pharmacological treatment.

## Materials and methods

### Study subjects

The current work was conducted within the framework of the CORDIOPREV study (Clinicaltrials.gov NTC00924937), a randomized, single-blinded, controlled dietary intervention trial including 1002 patients with CHD, who had their last coronary event more than six months before their enrolment in two different dietary models for seven years, in addition to following the conventional treatment for CHD [13]. The participants were randomized to receive two diets: a Mediterranean diet or a low-fat diet. Rationale, methodology and baseline characteristics of study participants have been published elsewhere [13]. The protocol was written in accordance with the principles of the Declaration of Helsinki. The respective Institutional Review Board (IRB) by the Human Investigation Review Committee of the Reina Sofia University Hospital (Córdoba, Spain) approved the study protocol. Recruitment took place from July 2009 to February 2012. All subjects provided written informed consent. The ethics approval and study protocol can be found on the IMIBIC website. At baseline and during every year of follow-up study participants passed medical and dietary interviews and underwent blood and oral glucose tolerance test (OGTT). The biochemical and laboratory measurements were performed as previously described [14, 15].

Patients who had diabetes diagnosed at the beginning of the study and were not receiving glucose-lowering treatment were included in the CORDIOPREV-DIRECT study (190 out of 1002 patients). The diabetes diagnosis was carried out according to the diagnosis criteria of the American Diabetes Association (ADA) (fasting glucose ≥ 126 mg/dL, 2 h glucose during OGTT ≥ 200 mg/dL, or HbA1c ≥ 6.5%) [16]. Of these, seven patients could not be included due to the inability to perform the diagnostic test used in this work. Since the aim of this sub-study were to evaluate changes in food and nutrients consumption from baseline to first year of follow-up, six more patients were excluded for not been disposable the food-frequency questionnaires (FFQ) at first year visit due to these patients denied continuing with dietary follow-up. Thus, for this sub-study, the final number of patients analyzed was 177.

### Criteria for diabetes remission

T2DM remission was defined as HbA1c < 6.5%, fasting plasma glucose < 126 mg/dL and 2 h plasma glucose after 75 gr OGTT < 200 mg/dL and maintaining these levels for at least 2 consecutive years without the use of diabetes medication to lower blood glucose levels [17, 18]. Patients were tested yearly from the first year of follow-up and classified as remission or maintaining diabetes at fifth year of the study. From the finally 177 patients included in the present work (those with biochemical tests and completed FFQ disposable), 72 patients reverted from T2DM after five years of dietary intervention, while 105 participants had not achieved remission by the end of the follow-up period.

### Study design and dietary assessment

The participants were randomized to receive two diets: a Mediterranean diet or a low-fat diet. The low-fat diet consisted of < 30% total fat (< 10% saturated fat, 12–14% monounsaturated fat (MUFA), and 6–8% polyunsaturated fat (PUFA)), 15% protein, and a minimum of 55% carbohydrates. The Mediterranean diet comprised a minimum of 35% of calories as fat (22% MUFA fat, 6% PUFA fat, and < 10% saturated fat), 15% proteins, and a maximum of 50% carbohydrates [13]. In both diets, the cholesterol content was adjusted to < 300 mg/d.

The dietary assessment has been described recently [19]. Participants in both intervention groups received the same intensive dietary counseling. Nutritionists carried out individual interviews at baseline and every six months, and quarterly group education sessions were held with up to 20 participants per session and separate sessions for each group. Information on habitual dietary intake was gathered by registered dietitians at baseline and at each year of follow-up using a validated 137-item semi-quantitative FFQ [20, 21]. To complete the FFQ, patients reported their average intake of different foods and beverages over the previous 12 months. Consumption frequencies were registered in nine categories ranging from “never or less than one time per month” to “six or more times per day”. Energy and nutrient intake were calculated using the Spanish Food Composition Tables [22, 23]. Percentage of contribution of foods and nutrients to the mean daily energy intake was also calculated. A more detailed description of the dietary intervention is shown in Quintana-Navarro et al. (2019) [19]. To investigate the association between changes in the consumption of plant-based proteins at the expense of proteins from animal source and T2DM remission, study participants were classified into two groups according to the median of change (Δ) in plant protein intake (median = 0.13%), expressed as percentage of energy (% E) (changes produced between post- and pre-intervention, calculated as the value of percentage of energy from plant protein after one year of dietary intervention minus the value at baseline), regardless of the type of consumed diet. Thus, patients whose Δ in plant-based protein consumption was below the median were sorted into the group which decreased plant protein intake (*n* = 89), and patients whose Δ in plant-based protein consumption was above the median were sorted into the group which increased plant protein intake (*n* = 88). With the former classification, it was possible to select study participants whose dietary habits were more noticeably modified towards a shift in plant protein intake after one year of follow-up thanks to dietary intervention.

### Laboratory analysis and anthropometric measurements

Following a 12 h fasting period, patients were admitted to the laboratory for anthropometric and biochemical test [body mass index (BMI), waist circumference, systolic blood pressure, diastolic blood pressure, HDL-cholesterol (c-HDL), LDL-cholesterol (c-LDL), triglycerides (TG), total cholesterol, highly sensitive C-reactive protein (hs-CRP), Alanine Aminotransferase (ALT), glucose, and HbA1c]. Smoking status, alcohol intake, and drug therapy were also registered for each participant.

Anthropometric parameters were measured by trained dietitians using calibrated scales (BF511 body composition analyzer/scale, OMROM, Japan) and a wall-mounted stadiometer (Seca 242, HealthCheck Systems, Brooklyn, NY). Waist circumference was measured midway between the lowest rib and the iliac crest. BMI was calculated as weight per square meter (kg/m2). Blood pressure was measured with a validated digital automated blood pressure monitor, and hypertension was defined as a systolic blood pressure ≥ 130 mmHg, diastolic blood pressure ≥ 85 mmHg, and/or current use of antihypertensive agents.

Venous blood samples were collected from the antecubital vein in Vacutainer ™ tubes containing EDTA or no anticoagulant. Serum parameters were measured by spectrophotometry using an Architect c-16000 analyser (Abbott^®^, Chicago, IL, USA): hexokinase method for glucose and oxidation-peroxidation for c-HDL, total cholesterol, and TG. C-LDL was calculated using the Friedewald formula provided serum TG levels were < 400 mg/dL. Plasma levels of insulin were measured by chemiluminescent microparticle immunoassay using an i-2000 Abbott Architect^®^ analyser. The plasma concentrations of hs-CRP were determined by high-sensitivity ELISA (BioCheck, Inc., Foster City, CA, USA).

### Statistical analyses

SPSS statistical software (IBM SPSS Statistics version 21.0) and R version 4.0.3 software (R Foundation for Statistical Computing, Vienna, Austria) [24] on the RStudio platform, and corrplot R package version 0.92 were used for statistical analysis of data. The normal distribution of variables was assessed using the Kolmogorov–Smirnov test. Data are represented as the mean ± SEM for continuous variables and as frequencies for categorical variables. *P* values ≤ 0.05 were considered statistically significant. The statistical differences in the metabolic variables between groups were evaluated by one-way ANOVA. Qualitative variables were compared using the Chi-square test. A repeated-measures ANOVA test was used to determine the statistical differences between variables at baseline and during the follow-up period. The post hoc statistical analysis was completed using Bonferroni's multiple comparison tests. Correlations between the Δ (1-year post-intervention minus baseline values) in energy, nutrients and food intake were calculated by means of a Spearman’s rank correlation.

Probability of T2DM remission was calculated using the Kaplan–Meier method of estimating the cumulative probability of an event in the groups of patients who had a Δ in plant protein (% E) intake above or below the median of the population after receiving dietary counselling (median = 0.13%). Time-dependent Cox regression models were used to identify significant factors associated with the time of incidence (full model was implemented with the following variables: sex, age, intervention group, baseline levels of c-HDL, TG, BMI, glucose, Hb1Ac, hs-CRP, insulin, and waist circumference, statin use, smoking status (never, former, or current smoker), alcohol intake, and familial history of diabetes).

## Results

### Baseline characteristic of the studied population

Characteristics of the subjects classified according to the median of Δ in plant protein consumption (expressed as % energy intake) at baseline are shown in Table 1. No statistically significant differences were observed in any of the anthropometric or clinical parameters. The proportion of men and women were differentially distributed among the two groups of Δ in plant protein intake with greater percentage of men in those who increased the plant protein intake (*p* = 0.034). Regarding the differences in energy, nutrients and food consumed at the beginning of the study (before initiating the dietary intervention), we found that those who increased the plant protein intake during the first years of follow-up, consumed more fat, MUFA, and PUFA, and less vegetal protein, carbohydrates, fiber, vegetables, cereals and derivates and whole grains at the baseline of the study (all *p* < 0.05) (Table supplementary 1).

**Table 1:** Baseline characteristics of study participants according to median of Δ in plant protein (%E) consumption

Variable	Decreased Plant Protein Intake (< P50 [− 3.21,0.13])	Increased Plant Protein Intake (> P50 (0.13,4.11])	*P* value
*n*	89	88	
Age (years)	59.6(1.0)	59.9(1.0)	0.866
Men/women (n)	70/19	79/9	0.034
Weight (Kg)	85.0	85.5	0.836
Waist circumference (cm)	105(1.1)	106(1.1)	0.621
BMI (kg/m2)	31.3(0.5)	31.1(0.4)	0.740
WHTR	0.64(0.007)	0.65(0.008)	0.248
TG (mg/dL)	158(10.0)	137(6.7)	0.085
Total cholesterol (mg/dL)	166(3.3)	162(3.5)	0.455
c-LDL (mg/dL)	91.9(2.8)	91.5(2.9)	0.915
c-HDL (mg/dL)	41.0(0.9)	41.1(1.1)	0.917
Apo A1 (mg/dL)	127(1.8)	126(2.4)	0.517
Apo B (mg/dL)	78.6(2.4)	74.3(1.8)	0.153
hs-CRP (mg/L)	3.05(0.26)	2.96(0.20)	0.787
Glucose (mg/dL)	110(2.4)	111(2.8)	0.737
HbA1c (%)	6.57(0.06)	6.80(0.10)	0.058
Insulin (mU/L)	11.7(1.3)	11.9(0.9)	0.898
HOMA-IR	4.51(0.47)	4.01(0.24)	0.341
ALT (U/L)	27.0(1.4)	29.7(1.4)	0.173
Treatment with statins, %	84.3	92.0	0.085
Hypertension, %	57.3	67.0	0.119
Current smoking, %	9.0	14.8	0.255
Prior smoking, %	60.7	58.0	0.761
Diet (LowFat/Med diet)	53/36	46/42	0.205

Values expressed as mean (SEM). *BMI* body mass index, *WHTR* Waist to height ratio, *TG* triglycerides, *c-HDL* high-density lipoprotein cholesterol, c-LDL low-density lipoprotein cholesterol, *Apo A1* Apolipoprotein *A1 Apo B *apolipoprotein B, *hs-CRP* high-sensitivity C-reactive protein, *HbA1c* glycosylated haemoglobin, *HOMA-IR* homeostasis model assessment-insulin resistance, *ALT* Alanine Aminotransferase, *Med diet* mediterranean diet. Continuous variables were analysed using t-test or Wilcoxon rank sum test for unpaired data when data did not fit the normal distribution. Categorical variables were analysed using *χ*^2^ test

### Probability of T2DM remission

**Fig. 1:**
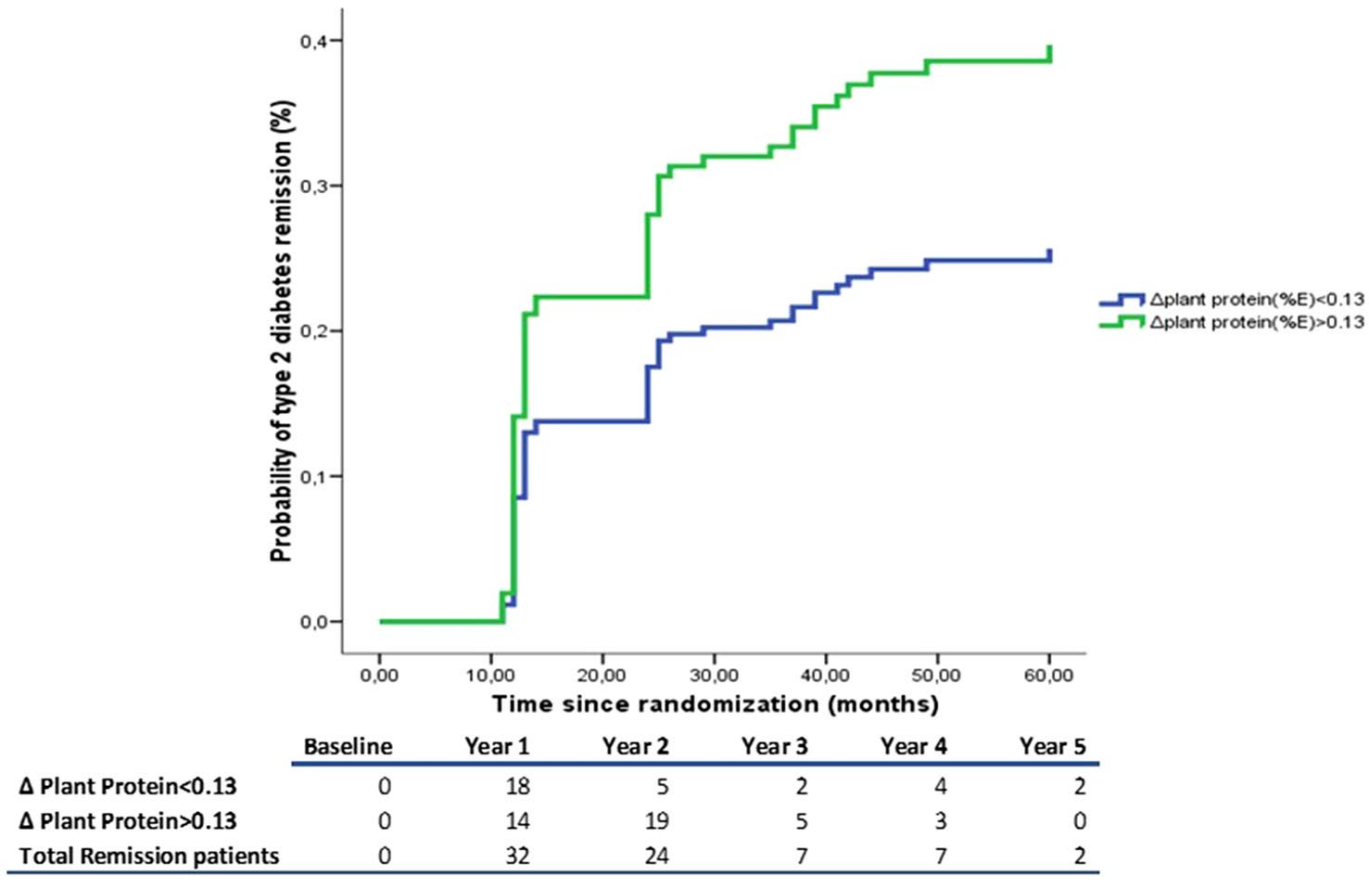
Probability of type 2 diabetes remission. Probability of type 2 diabetes remission according to Δ plant protein intake (< 0.13%, those who decreased the plant protein consumption; > 0.13%, those who increased the plant protein consumption). The model was adjusted for baseline levels of HDL, triglycerides, BMI, glucose, Hb1Ac, PCR, Insulin, and waist circumference, statin use, smoking status (never, former or current smoker), alcohol intake and familial history of diabetes. The HR between groups was calculated. Table adjacent represent the number of remissions per year of follow-up

The probability of T2DM remission depending on the Δ of plant protein intake during dietary intervention was estimated using a Kaplan–Meier survival curve (Fig. 1). To that end, patients were classified according to median in Δ plant protein intake (first year of follow-up vs baseline). We observed in the table adjacent to survival curve that majority of T2DM remission occurred at first and second year of follow-up, and that the number of patients that achieved remission in the third year onwards diminished compared to previous years. We also found that patients who increased plant protein intake had higher probability of T2DM remission than those who decreased plant protein intake, after adjustment with a hazard ratio (HR) of 1.71 (1.05–2.77) (*p* = 0.031). Hazard ratios for T2DM remission according to the groups studied are presented in Table 2.

**Table 2:** HRs (95% CIs) of T2DM remission according to the median of Δ plant protein (%E) consumption

	Increased Plant Protein Intake (> P50 (0.13,4.11])	Likelihood ratio test
Unadjusted model	1.453(0.911–2.318)	*p* = 0.117
Multivariable model 1	1.453(0.911–2.318)	*p* = 0.117
Multivariable model 2	1.706 (1.050–2.772)	*p* = 0.031

Cox regression models were used to assess the risk of T2DM according to median of Δ plant protein (%E) consumption. Multivariable model 1 was adjusted for age, sex and intervention group. Model 2 was further adjusted for baseline levels of HDL, triglycerides, BMI, glucose, Hb1Ac, PCR, Insulin, and waist circumference, statin use, smoking status (never, former or current smoker), alcohol intake and familial history of diabetes

### Plant protein intake after dietary intervention

**Fig. 2:**
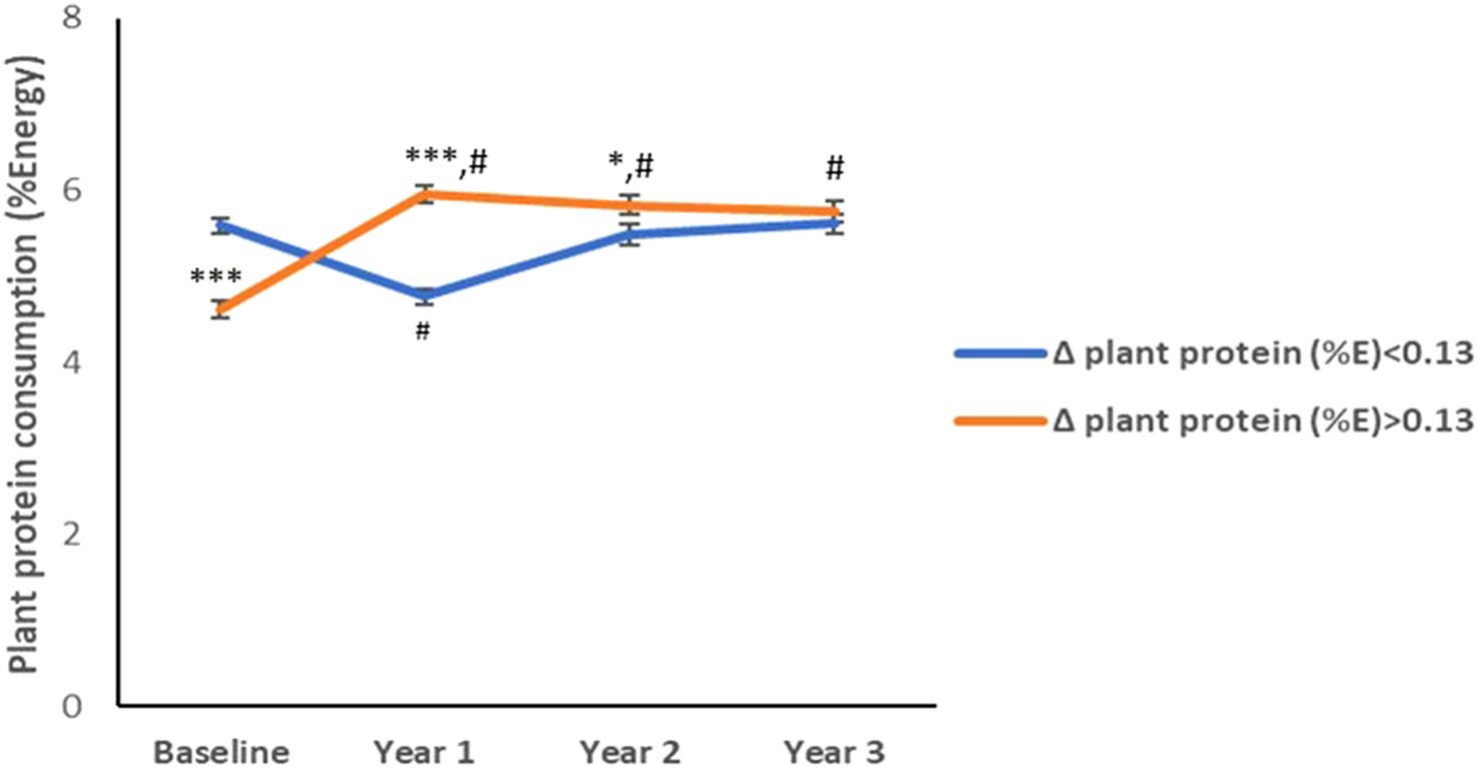
Follow-up plant protein consumption. Average plant protein consumption during 3 years of follow-up. Values are expressed as mean ± SEM. ***p < 0.001, **p < 0.01, and *p < 0.05 for comparisons between groups at each visit. #*p* < 0.001 for comparisons with baseline in each group. Δ, change

Participants belonging to the group of patients who increased plant protein intake significantly modified the amount of plant protein in their diet compared to baseline and maintained the changes for at least 3 years of follow-up (Fig. 2). Patients who decreased the plant protein intake modified the amount of plant protein in its diet compared to baseline only until the first year of follow-up, after that they maintained the amount of plant protein they consumed at baseline. However, the amount of plant protein intake by the group who increased their consumption was maintained higher than those consumed by the other group during the follow-up until the third year (Fig. 2).

### Changes in energy, nutrients, and food intake

Table 3 summarizes mean changes in energy, nutrients, and food intake, according to the data collected in the FFQs, as the values at first year of follow-up minus the baseline. In the two studied groups, modification of baseline dietary habits led to a significant reduction in total energy, cholesterol, and SFA (expressed as % of total fat), and increment in PUFA (%total fat), and fruit consumption. On the other hand, those who increased the plant protein intake had reduction in fat, mainly SFA (%E) and MUFA (%E) but without changes as expressed in %total fat, and animal protein consumption, and significant increment in plant protein, carbohydrates, fiber, vegetables, legumes, tree nuts, cereals, and whole grains intake. Furthermore, those patients who decreased the plant protein intake reduced the plant protein, carbohydrates, tree nuts and cereals intake with increment in animal protein consumption. A more detailed description of the changes in nutrients and food intake along the follow-up can be found in Quintana-Navarro et al. [19].

**Table 3:** Delta Changes after 1 year of intervention values in energy, nutrient, and food intake

Variable	Decreased Plant Protein Intake (< P50 [− 3.21,0.13]) *n* = 89	Increased Plant Protein Intake (> P50 (0.13,4.11]) *n* = 88	Between-group differences post-intervention (*p* value)
Energy, kcal/d	− 508(62)*	− 443(54)*	0.433
Fat (%E)	0.94(0.72)	− 4.54(0.85)*	< 0.001
SFA (%E)	− 0.32(0.21)	− 2.06(0.23)*	< 0.001
SFA (% total fat)	− 1.47(0.43)*	− 2.78(0.44)*	0.034
MUFA (%E)	0.32(0.50)	− 2.18(0.55)*	0.001
MUFA (% total fat)	− 0.54(0.83)	0.13(0.72)	0.543
PUFA (%E)	1.20(0.37)*	0.18(0.26)	0.025
PUFA (% total fat)	2.94(0.88)*	2.80(0.63)*	0.893
Protein (%E)	0.22(0.37)	0.14(0.38)	0.880
Vegetal protein (%E)	− 0.82(0.08)*	1.34(0.10)*	< 0.001
Animal protein (%E)	1.03(0.39)*	− 0.94(0.40)*	< 0.001
Carbohydrates (%E)	− 2.18(0.74)*	4.26(0.88)*	< 0.001
Cholesterol (mg/d)	− 75.5(11.9)*	− 109.7(10.1)*	0.030
Fibre, g/1000 kcal	0.59(0.39)	3.45(0.41)*	< 0.001
Fruit, g/1000 kcal	56.6(13.8)*	36.2(11.7)*	0.265
Vegetables, g/1000 kcal	9.51(6.19)	30.64(8.11)*	0.040
Legumes, g/1000 kcal	− 0.10(0.75)	3.26(1.17)*	0.017
Tree nuts, g/1000 kcal	− 1.32(0.52)*	1.01(0.72)*	0.010
Cereals and derivatives, g/1000 kcal	− 26.1(3.06)*	18.75(3.76)*	< 0.001
Whole grains, g/1000 kcal	2.90(4.40)	22.98(4.93)*	0.003

Values are expressed as mean (SEM). *SFA* saturated fatty acids, *MUFA* monounsaturated fatty acids; *PUFA* polyunsaturated fatty acids. Between-group differences were assessed using *t*-test or Wilcoxon rank sum test for unpaired data when data did not fit normal distribution, and within-group differences were assessed using paired *t*-test or Wilcoxon signed rank test when data did not fit normal distribution. Within-group differences from baseline: *p < 0.05

### Changes in biochemical and anthropometrics parameters

We evaluated changes in biochemical and anthropometrics parameters after the first year of follow-up in both groups of patients studied. We found that those patients who increased the plant protein consumption reduced the waist to height ratio (WHTR, a biomarker of ectopic fat deposit), and HbA1c, whilst no changes were found in those patients who decreased the plant protein intake. On the other hand, both groups of patients decreased the levels of ALT and TG, but with greater reduction in the group of patients who decreased plant protein consumption (Table 4).

**Table 4:** Delta Changes after 1 year of intervention values in biochemical and anthropometrics parameters

Variable	Decreased Plant Protein Intake (< P50 [− 3.21,0.13]) *n* = 89	Increased Plant Protein Intake (> P50 (0.13,4.11]) *n* = 88	Between-group differences post-intervention (p value)
Weight (kg)	− 0.53(2.00)	1.61(2.10)	0.461
Waist circumference (cm)	− 1.42(1.58)	0.13(1.75)	0.512
BMI (kg/m2)	− 0.83(0.63)	0.30(0.68)	0.221
WHTR	− 0.005(0.003)	− 0.010(0.004)*	0.306
TG (mg/dL)	− 30.1(8.9)^**#**^	− 4.39(7.51)	0.029
Total cholesterol (mg/dL)	− 4.67(3.19)	-1.14(3.24)	0.439
c-LDL (mg/dL)	3.48(3.45)	2.57(2.73)	0.835
c-HDL (mg/dL)	1.14(0.86)	0.21(0.69)	0.399
Apo A1 (mg/dL)	− 2.02(2.04)	-2.66(1.73)	0.811
Apo B (mg/dL)	− 5.42(2.00)*	− 5.43(1.85)*	0.999
hs-CRP (mg/L)	1.67(1.65)	0.38(0.42)	0.452
Glucose (mg/dL)	− 2.79(2.04)	− 0.62(1.85)	0.434
HbA1c (%)	− 0.03(0.08)	− 0.35(0.08)^**#**^	0.006
Insulin (mU/L)	− 3.40(1.28)*	− 2.79(1.02)*	0.711
HOMA-IR	− 2.09(0.54)^**#**^	− 1.44(0.20)^**#**^	0.244
ALT (U/L)	− 2.56(1.23)*	− 3.44(1.14)*	0.604

Values are expressed as mean (SEM). *BM*I body mass index, *WHTR *waist to height ratio, *TG* triglycerides, *c-HDL* high-density lipoprotein cholesterol, *c-LDL* low-density lipoprotein cholesterol, *Apo A1* apolipoprotein A1, *Apo B* apolipoprotein B, *hs-CRP* high-sensitivity C-reactive protein, *HbA1c* glycosylated haemoglobin, *HOMA-IR* homeostasis model assessment-insulin resistance, *ALT* alanine Aminotransferase. Between-group differences were assessed using *t*-test or Wilcoxon rank sum test for unpaired data when data did not fit normal distribution, and within-group differences were assessed using paired *t*-test or Wilcoxon signed rank test when data did not fit normal distribution. Within-group differences from baseline: *p < 0.05; #*p* < 0.001

### Correlations between changes in energy, nutrients, and food intake

**Fig. 3:**
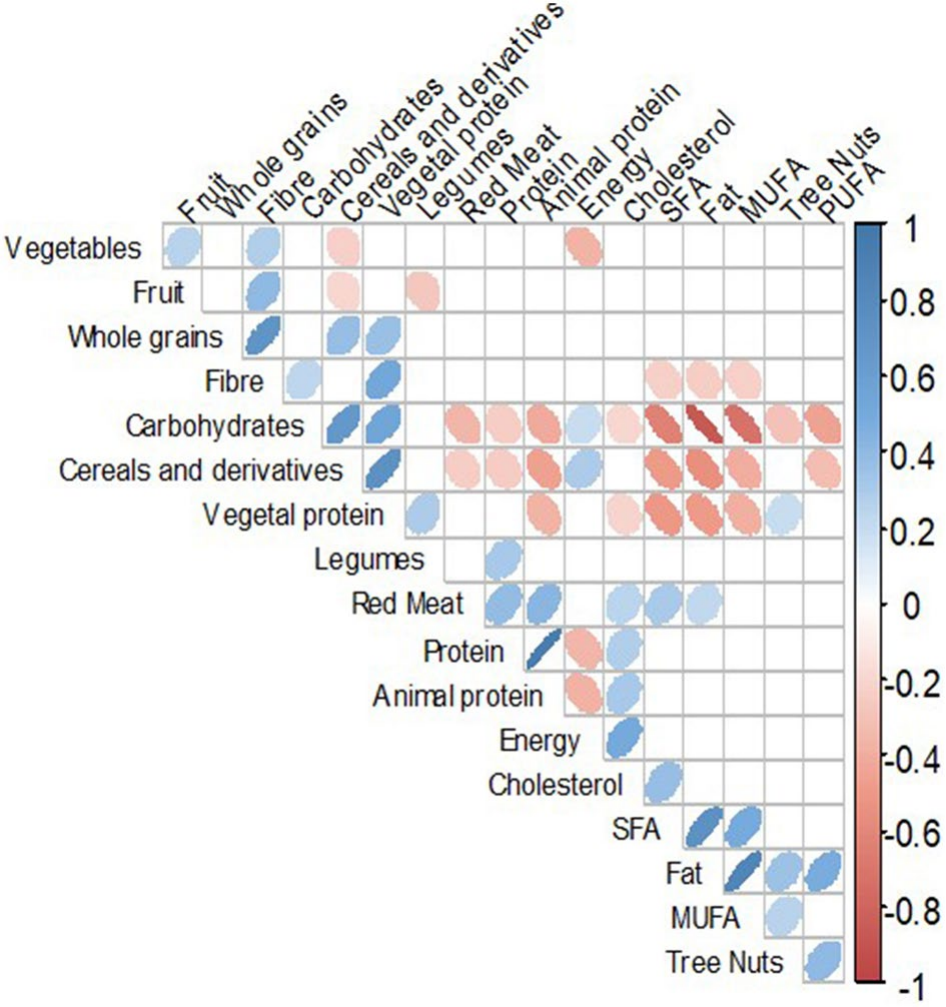
Correlogram of the upper triangular of the correlation matrix of the change in energy, nutrients, and food consumption (adjusted by energy) of 177 individuals from the CORDIOPREV (coronary diet intervention with olive oil and cardiovascular prevention) study after receiving dietary counselling. Figure shows correlations which *p* values were < 0.01. Positive correlations are displayed in blue and negative correlations in red, and colour intensity is proportional to the correlation coefficient. The ellipses have their eccentricity parametrically scaled to the correlation value. *SFA* saturated fatty acids, *MUFA* monounsaturated fatty acids, and *PUFA* polyunsaturated fatty acids

To assess the interrelation between changes in dietary variables adjusted by energy, a Spearman correlation matrix was used (Fig. 3). Changes in plant protein intake were positively correlated with changes in the intake of carbohydrates, fiber, legumes, tree nuts, cereals and derivatives, and whole grains, and negatively correlated with changes in the intake of SFA, MUFA, and total fat, cholesterol, and animal protein.

## Discussion

In this study, we demonstrated that increments in plant protein intake are associated with a higher probability of T2DM remission, in the context of a dietary intervention without caloric restriction nor glucose-lowering treatment, thus describing for the first time the impact of a particular nutrient on the probability of T2DM remission in newly diagnosed diabetes patients with CHD.

During the last decade, the efforts of the scientific community have been directed towards the study of diabetes remission, overturning the belief that it was a lifelong and progressive condition [25]. T2DM remission have been mainly associated with weight loss achieved by bariatric surgery or by hypocaloric diets [7, 8]. However, to the best of our knowledge, none of the published studies have analyzed dietary components to identify nutrients directly associated with diabetes remission. In the context of CORDIOPREV study, our research group has described a set of newly diagnosed T2DM patients who achieved diabetes remission after consumption of two healthy dietary models (Mediterranean or low-fat diet) without receiving glucose-lowering therapy. We have already reported that physiological status regarding insulin resistance and beta-cell functionality determined the remission in this population, showing that pancreatic function, measured by Disposition Index (DI) [10], was the most important factor. Although dietary intervention did not show any between-diet differences, since both diets presented similar remission rates, Mediterranean diet seemed to induce a reduction in circulating levels of advanced glycation end products (AGEs, molecules with highly oxidant and proinflammatory properties) that favor the likelihood of T2DM remission [9]. However, we wanted to go further and seek for nutritional components shared by these two diets that could affect T2DM remission.

High-protein diets help to prevent and manage T2DM, as proteins exhibit insulinotropic effects. In this sense, long-term studies on the effects of high-protein diets have reported that increases in dietary proteins, especially from animal sources, are positively associated with T2DM development [26–28], whereas plant-based proteins are negatively associated [29]. A meta-analysis of randomized controlled trials suggested replacing animal for plant-based proteins had beneficial effects on glycemic control in individuals with T2DM [11]. A different meta-analysis reported the type and abundance of specific amino acids, differentially distributed among animal and plant proteins, might contribute to T2DM risk [29]. In this sense, animal proteins contain higher levels of branched chain amino acids in comparison with plant proteins, and leucine, isoleucine and methionine may be risk factors for insulin resistance and T2DM [29]. In this regard, we have recently demonstrated that T2DM-free patients who increased plant protein consumption presented less likelihood of diabetes onset after 5-year of follow-up [12].

Notwithstanding diabetes remission is a recent concept, there are several studies investigating the molecular mechanisms, clinical aspects and lifestyle behaviors influencing this phenomenon. Regarding dietary intervention studies, most of them have been focused on the effect of drastic weight loss. For instance, the DiRECT study showed that people who achieved T2DM remission lost 16.2 ± 1.2 kg after a dietary intervention with a low-calorie-diet based on liquid formula followed by a weight maintenance diet [7, 8]. CORDIOPREV dietary intervention is based on two healthy dietary patterns, which helps to improve dietary adherence in a free-living way of life with the intention to treat, better than a liquid formula diet [19]. Besides the increasing number of studies, to the best of our knowledge there are no publications investigating protein sources in relation to diabetes remission. In the context of the management of T2DM patients, Satija et al.suggested a healthful plant-based diet index conferring positive scores to the intake of whole grains, vegetables, fruits, nuts, pulses, and vegetable oils, and negative ones to the intake of less healthy plant-based foods, such as fruit juices or refined grains [30]. These findings support the need of comprehensive nutritional advice towards diets rich in healthy plant-based foods. In our study, both dietary interventions (low-fat and Mediterranean diets) are considered healthy cardioprotective dietary patterns that promote diminishing SFA and increasing fiber and high-complexed carbohydrates.

CORDIOPREV study patients received comprehensive nutritional advice aiming the overall quality of the diet, which is an advantage over the aforementioned studies, where intervention was carried out with a liquid formula or diet directly supplied by researchers [8, 31]. In contrast, nutritional counselling provided by CORDIOPREV dietitians, although did not aim to specific changes in plant protein consumption, it encouraged the intake of legumes, whole grains, nuts (in Mediterranean diet), and vegetables, while discouraging the consumption of red or processed meat [19]. In terms of protein consumption, we found a significant increment in the percentage of energy obtained from plant proteins up to an average of 6%, regardless of dietary pattern. This was in accordance with Zhao et al. who reported the largest risk reduction of T2DM when energy intake from plant proteins was about 6% [32].

On the other hand, when studying health effects of plant protein intake, we must consider food matrix as a whole. In this sense, plant-based protein is consumed with abundant dietary fiber and micronutrients, which makes it complex to attribute health benefits to the single nutrient studied [33]. In the general population, plant or animal protein intake has been associated with diet quality, with plant protein intake being described as a robust marker of diet nutrient adequacy [34, 35]. Our results support the idea of the synergistic effect of dietary components, as we found that an increase in the consumption of animal protein was positively correlated with cholesterol and total protein, and plant protein was positively correlated with fiber, legumes intake, carbohydrates, and whole grains, and strongly negatively correlated with cholesterol, fat, and SFA.

The effects of dietary components on pathophysiological pathways involved in glycemic control and insulin actions have been extensively discussed in several publications [36–38]. In this regard, dietary fiber interferes with carbohydrate and protein absorption reducing postprandial glucose response. Similarly, replacement of animal protein with protein from plant sources reduces organism supply of heme iron, levels of AGEs, cholesterol, nitrate and nitrite, trimethylamine N-oxide, and branched chain amino acids, all of which have been associated with T2DM development. However, the role of plant proteins per se in metabolic pathways related to T2DM is not that clear. For instance, some authors have associated the reduction in T2DM risk with an improvement of body weight, blood pressure, blood lipids profile and inflammatory biomarkers [39]. Dietary effects leading the remission, mainly, based on weight loss which reduces ectopic fat deposit in liver and pancreas, have been associated with reduction in HbA1c and liver damage markers such as ALT [4, 37]. Accordingly, we found a reduction in the percentage of HbA1c, ALT and WHTR (a biomarker of visceral fat deposit) in those patients who increased the plant protein consumption.

The major strength of this study is that this is the first approach to assess T2DM remission according to changes in a particular nutrient administrating two healthy diets with no caloric restriction, weight loss, or any pharmacological treatment in patients with CHD. However, we observed that the number of patients that achieved remission in the third year onwards diminished compared to previous years. We could hypothesize that a possible reason for this apparent loss of effect on other factors such as the time of course of the disease, perhaps patients in the initial stages of the disease are more susceptible to respond to changes in the diet. Moreover, some limitations should be highlighted. This research is based on a long-term, well-controlled dietary intervention, which ensures the quality of the study, but might not reflect the level of compliance in a free-living population. Secondly, T2DM remission was not the primary endpoint of the CORDIOPREV trial, but a secondary objective of this study, what make no possible to link causality from our observations. Thirdly, even though there is limited long-term study regarding dietary intervention, ours comprises 60 months of follow-up, the sample size in our study should be considered as a limitation. Finally, we have to mention that did not include physical activity as a possible confounding factor.

In conclusion, the results of the present study support the need to improve the quality of the diet, including dietary proteins, increasing the consumption of those of vegetal origin as dietary therapy to reverse T2DM in the context of healthy diets recommended for CHD patients.

## Supplementary Information

Below is the link to the electronic supplementary material.

Supplementary file1 (DOCX 16 KB)

## Data Availability

The data that support the findings of this study are available from the corresponding author upon reasonable request.
