# Supplementary material for: Changes in quantity plant-based protein intake on type 2 diabetes remission in coronary heart disease patients: from the CORDIOPREV study
Source: Eur J Nutr. 2023 Mar 4;62(4):1903–13. doi: 10.1007/s00394-022-03080-x (PMC10195707; doi:10.1007/s00394-022-03080-x)
Supplement: Supplementary file 1 — Supplementary file1 (DOCX 16 KB) [file 394_2022_3080_MOESM1_ESM.docx]

**Table supplementary 1**. Baseline values of energy, nutrients, and food according to median of Δ in plant protein (%E) consumption.

| **Variable** | **Baseline** | | |
| --- | --- | --- | --- |
|  | **Decreased Plant**  **Protein Intake**  **(<P50 [−3.21,0.13]) n=89** | **Increased Plant**  **Protein Intake**  **(>P50 (0.13,4.11])**  **n=88** | **between-group  differences  (p-value)** |
| **Energy, kcal/d** | 2.380(61) | 2.315(56) | 0.435 |
| **Fat (%E)** | 35.5(0.5) | 38.1(0.6) | **0.001** |
| **SFA (%E)** | 8.71(0.17) | 9.15(0.21) | 0.106 |
| **SFA (% total fat)** | 24.6(0.4) | 24.0(0.4) | 0.292 |
| **MUFA (%E)** | 17.0(0.3) | 19.0(0.4) | **<0.001** |
| **MUFA (% total fat)** | 47.9(0.5) | 49.6(0.5) | **0.011** |
| **PUFA (%E)** | 6.27(0.18) | 6.29(0.17) | 0.919 |
| **PUFA (% total fat)** | 17.7(0.4) | 16.5(0.4) | **0.047** |
| **Protein (%E)** | 18.6(0.3) | 18.2(0.3) | 0.360 |
| **Vegetal protein (%E)** | 5.57(0.09) | 4.62(0.09) | **<0.001** |
| **Animal protein (%E)** | 12.3(0.3) | 12.8(0.3) | 0.241 |
| **Carbohydrates (%E)** | 43.8(0.6) | 40.9(0.7) | **0.001** |
| **Cholesterol (mg/d)** | 338(13) | 344(11) | 0.741 |
| **Fibre, g/1000 Kcal** | 12.2(0.4) | 10.6(0.3) | **0.001** |
| **Fruit, g/1000 Kcal** | 153(10) | 177(10) | 0.104 |
| **Vegetables, g/1000 Kcal** | 127(5) | 103(5) | **0.001** |
| **Legumes, g/1000 Kcal** | 11.3(0.7) | 10.6(0.6) | 0.418 |
| **Tree nuts, g/1000 Kcal** | 4.42(0.55) | 3.33(0.47) | 0.132 |
| **Cereals and derivatives, g/1000 Kcal** | 90.8(2.6) | 70.1(2.9) | **<0.001** |
| **Whole grains, g/1000 Kcal** | 26.1(3.8) | 12.7(2.6) | **0.005** |

Values are expressed as mean (SEM). SFA: saturated fatty acids; MUFA: monounsaturated fatty acids; PUFA: polyunsaturated fatty acids. Continuous variables were analysed using t-test or Wilcoxon rank sum test for unpaired data when data did not fit the normal distribution.
